# Structure-Activity Relationships of FMRF-NH_2_ Peptides Demonstrate A Role for the Conserved C Terminus and Unique N-Terminal Extension in Modulating Cardiac Contractility

**DOI:** 10.1371/journal.pone.0075502

**Published:** 2013-09-17

**Authors:** Benjamin F. Maynard, Chloe Bass, Chris Katanski, Kiran Thakur, Beth Manoogian, Megan Leander, Ruthann Nichols

**Affiliations:** 1 Department of Biological Chemistry, The University of Michigan Medical School, Ann Arbor, Michigan, United States of America; 2 Undergraduate Biochemistry Honors Research Program, Department of Chemistry, The University of Michigan, Ann Arbor, Michigan, United States of America; 3 Undergraduate Chemistry Honors Research Program, Department of Chemistry, The University of Michigan, Ann Arbor, Michigan, United States of America; University of South Florida College of Medicine, United States Of America

## Abstract

FMRF-NH_2_ peptides which contain a conserved, identical C-terminal tetrapeptide but unique N terminus modulate cardiac contractility; yet, little is known about the mechanisms involved in signaling. Here, the structure-activity relationships (SARs) of the *Drosophila melanogaster* FMRF-NH_2_ peptides, PDNFMRF-NH_2_, SDNFMRF-NH_2_, DPKQDFMRF-NH_2_, SPKQDFMRF-NH_2_, and TPAEDFMRF-NH_2_, which bind FMRFa-R, were investigated. The hypothesis tested was the C-terminal tetrapeptide FMRF-NH_2_, particularly F1, makes extensive, strong ligand-receptor contacts, yet the unique N terminus influences docking and activity. To test this hypothesis, docking, binding, and bioactivity of the C-terminal tetrapeptide and analogs, and the FMRF-NH_2_ peptides were compared. Results for FMRF-NH_2_ and analogs were consistent with the hypothesis; F1 made extensive, strong ligand-receptor contacts with FMRFa-R; Y→F (YMRF-NH_2_) retained binding, yet A→F (AMRF-NH_2_) did not. These findings reflected amino acid physicochemical properties; the bulky, aromatic residues F and Y formed strong pi-stacking and hydrophobic contacts to anchor the ligand, interactions which could not be maintained in diversity or number by the small, aliphatic A. The FMRF-NH_2_ peptides modulated heart rate in larva, pupa, and adult distinctly, representative of the contact sites influenced by their unique N-terminal structures. Based on physicochemical properties, the peptides each docked to FMRFa-R with one best pose, except FMRF-NH_2_ which docked with two equally favorable poses, consistent with the N terminus influencing docking to define specific ligand-receptor contacts. Furthermore, SDNAMRF-NH_2_ was designed and, despite lacking the aromatic properties of one F, it binds FMRFa-R and demonstrated a unique SAR, consistent with the N terminus influencing docking and conferring binding and activity; thus, supporting our hypothesis.

## Introduction

Peptidergic signaling plays critical roles in transmitting messages and in regulating physiological function. Thus, it is important to delineate the mechanisms associated with peptidergic signaling to identify the molecules involved in order to influence activity. Physiologically active peptides can often be grouped together based on a common structural motif and, frequently, functionally similar orthologous structures exist in vertebrates and invertebrates. This high degree of structural and functional conservation is consistent with the orthologs playing critical roles in physiology. The conservation also suggests research conducted in a lower, more experimentally versatile organism is a powerful approach to gain insight into the role peptides play in transmitting messages and regulating physiological function across phylogeny.

Members of one peptide family are structurally related by an identical RF-NH_2_ C terminus and found throughout the animal kingdom. The first member of this peptide family identified was the molluscan cardioexcitatory tetrapeptide FMRF-NH_2_ [1]. Subsequently, numerous structurally-related peptides were identified in both vertebrates and invertebrates and termed FaRPs, FMRF-NH_2_-related peptides. The members of this superfamily all contain RF-NH_2_; however, the peptides can be further divided into subgroups based on the structure of the C-terminal tetrapeptide, primarily driven by the identity of the amino acid in position 1. For instance, *Drosophila melanogaster* FMRF-NH_2_ defines one subgroup of FaRPs, yet, the role of F1 in the structure-activity relationship (SAR) of the subgroup remains unanswered; HMRF-NH_2_ defines another subgroup. Vertebrate neuropeptide FF (NPFF) and cholecystokinin (CCK) are orthologs of these two invertebrate peptide subgroups, respectively [2]–[4].

Peptides with an identical C-terminal tetrapeptide FMRF-NH_2_ but a unique N-terminal structure are abundant messengers present in neural tissue which act to modulate cardiac contractility. However, much remains to be discovered about the mechanisms by which these structurally-related peptides influence physiological function. In particular, the roles that the highly conserved C-terminal tetrapeptide and the unique N-terminal structures play in conferring activity is relatively uninvestigated, yet, this information provides data needed to delineate and, thus, target mechanisms underlying crucial physiological processes.

Typically, within a single species, numerous FaRPs exist which are produced from multiple, distinct precursors. In addition, the structurally-related peptides may be encoded in polyproteins which undergo processing to release several gene products. The *D. melanogaster* FMRF-NH_2_ (dFMRFa) gene encodes PDNFMRF-NH_2_, SDNFMRF-NH_2_, DPKQDFMRF-NH_2_, SPKQDFMRF-NH_2_, and TPAEDFMRF-NH_2_ [5], [6]. The endogenous, naturally-occurring dFMRFa peptides were identified and their structures confirmed [5], [7], [8]. In addition, the peptides were found to bind to one G protein-coupled receptor (GPCR) designated FMRFa-R [9], [10]. The first report of FMRF-NH_2_-like material in *D. melanogaster* identified immunoreactive material distributed on a developmental level in the central nervous system [11]. The antisera used, however, recognized an antigen, RF-NH_2_, present in many structurally-related peptides. Subsequently, N-terminal specific antisera were generated to the five FMRF-NH_2_-containing peptides encoded in dFMRFa to individually map their cellular expression [12]. The distinct, non-overlapping cellular expression patterns of the five FMRF-NH_2_-containing peptides suggest they play multiple, diverse roles in physiology and/or receive disparate input which regulates their release and effects.

The SARs of DPKQDFMRF-NH_2_, SPKQDFMRF-NH_2,_ PDNFMRF-NH_2_, SDNFMRF-NH_2_, and TPAEDFMRF-NH_2_, encoded in dFMRFa were a focus of this study in order to gain insight into how these structurally-related peptides act. The hypothesis tested was the C-terminal tetrapeptide FMRF-NH_2_, particularly F1, makes strong and extensive ligand-receptor contacts, yet the unique N terminus influences docking and activity. This prediction was based on several facts including the strict conservation of a C-terminal tetrapeptide in multiple peptides within a single species and across phylogeny. To test whether FMRF-NH_2_, particularly F1, makes numerous, strong ligand-receptor contacts the C-terminal tetrapeptide and analogs were docked to FMRFa-R and the results were compared to whether the peptide bound to expressed receptor protein. Additionally, the widespread presence and processing of FMRF-NH_2_-likeextended peptides from single precursors across the animal kingdom supports this hypothesis. The existence of only one receptor to which the multiple FMRF-NH_2_-containing peptides bind is also consistent with this prediction. Docking and bioactivity were investigated and compared to binding for the *D. melanogaster* FMRF-NH_2_-containing peptides to test whether the unique N-terminal extensions conferred diversity in the contact sites for the multiple ligands interacting with one receptor. Finally, an analog of a FMRF-NH_2_ peptide with the substitution replacing an amino acid predicted to be involved in docking and binding was analyzed to investigate the influence of the N-terminal extension in docking and activity, and test our hypothesis.

## Materials and Methods

### Ethics statement

This research utilized an invertebrate model, thus, no experimental procedure required an animal care and use review.

### Animals

*D. melanogaster* Oregon R strain flies were maintained on cornmeal molasses media at 24°C under a 12 hour light/dark cycle. Animals analyzed were wandering 3^rd^ instar larvae, white prepupae, and 1 day adults. The effects of peptides and physiological saline, the carrier, were measured on both females and males; no sex-specific response was observed.

### Chemicals

All peptides were synthesized on a 433A Applied Biosystems peptide synthesizer using standard Fmoc procedures and purified by reversed-phase high performance liquid chromatography. Each synthesis was obtained with ≥95% purity and identified by matrix-assisted laser desorption/ionization time-of-flight mass spectrometry. Peptides were diluted in series with physiological saline to obtain the working solutions.

### Bioassays

The *in vivo* semi-isolated heart rate assays were performed according to a previously reported protocol [13]. The rate of heart contractions for each animal was measured prior to application of a peptide (1 µM) or saline, the carrier, to provide a measure of basal rate. Animals (n≥6) were used only once. During and after delivery of the peptide or saline, contractions were continuously recorded for a 10-minute time period.

### Data analysis

The data analyzed were the maximum responses within the 10-minute recording period reported as mean values ± S.E.M. Data are reported as % basal heart rate where the frequency of contractions before application of peptide or saline is considered a measure of the baseline or basal level. Effects of peptides were compared to saline using Single Factor ANOVA with significance set at p ≤ 0.01; a peptide was considered to be active if its effects were statistically different from saline. The statistical outcomes were identical when calculated using two different data packages, Microsoft Excel and IBM SPSS.

### Receptor and ligand modeling

The primary sequence of FMRFa-R (Accession # AAL83921.1) was modeled by GPCR I-TASSER (zhanglab.ccmb.med.umich.edu) [14], [15] to generate a three-dimensional receptor structure [16]. The top scoring model was refined in ModRefiner (zhanglab.ccmb.med.umich.edu) [17], which uses backbone topology, hydrogen bonds, and side chain interactions to bring the model closer to an energetic minimum. The refinement output contained side chains in energetically favorable positions with steric clashes resolved. After the receptor structure was viewed in the PyMOL Molecular Graphics System, version 1.5.04 (Schrödinger, LLC) the extracellular loops (ECLs) and tails were removed. The high degree of flexibility and structural variation even between related receptors in the tail and loop regions often prevents accurate modeling and, thus, these structures are typically removed before ligand docking [18]–[20].

The refined receptor structure was submitted to binding site prediction at SiteHound (scbx.mssm.edu) and Q-SiteFinder (modelling.leeds.ac.uk) using methyl carbon, aromatic carbon, and hydroxyl oxygen probes [21], [22]; predicted characteristics of regions within the ligand binding pocket were considered in docking analysis. The receptor model was modified to include polar hydrogen atoms and partial charges where present, and a grid box that outlined the entire predicted ligand binding pocket to define the docking boundaries was selected with AutoDockTools v 4.2 [23]. Grid box size did not exceed 27000 cubic angstroms (Ås), the upper limit defined by AutoDock Vina [24]. Ligand models were built in PyMOL and also prepared for docking with AutoDockTools. Preparation for docking included defining rotatable bonds, assignment of partial charges and aromatic carbons, and removal of non-polar hydrogen atoms.

### Ligand docking

The molecular docking software AutoDock Vina [24] was used to predict putative ligand-receptor contact sites. Docking was run with AutoDock Vina at default exhaustiveness, roughly defined as the time invested in each docking run [24], and with the grid boxes as described above. Ligands were given conformational flexibility although receptors remained rigid. The number of binding modes (poses) generated was set to 20 for each run, and 10 runs were performed with each ligand-receptor pair, for a total of 200 docked ligand poses each. Running AutoDock Vina multiple times per ligand-receptor pair helped rule out the influence of the starting position on the poses produced by generating different random seeds and starting poses, and increased the quality of the results; a similar method was previously employed to investigate peptide docking [25].

### Docking analysis

AutoDock Vina predicts the binding affinity of each pose as an energy value in kcal/mol. Before poses were analyzed, affinities were scanned to ensure no results had positive values, which would indicate a run with poor results. Binding affinities were not considered further because no correlation between binding affinity values and the most favorable poses was observed. Further, binding affinity computations are not very accurate because they do not account for entropy and other important factors like solvation [26].

An overlay of the poses generated for a particular ligand-receptor docking pair was then visually examined in PyMOL. Two investigators independently and blindly analyzed the 200 poses, dividing them into groups of repeated results. Poses lacking similarity to others were not considered for further analysis. This approach lends itself to determining which output is the best docked because such a pose should be predicted from multiple starting seeds and occur more than once, therefore decoys are excluded from analysis. Several criteria including physicochemical side chain properties and type and proximity of ligand-receptor contact sites were used to evaluate groups to identify the most favorable docked conformation(s).

Poses were surveyed to identify favorable ligand-receptor interactions as measured by the physicochemical properties of the side chains. Distances for strong interactions met the following criteria: hydrogen bonds, 2.2–4Å [27]; cation-pi bonds, 2.9–3.6Å [28]; pi-stacking interactions, 3.5–7.5Å [29]; hydrophobic interactions, <5Å [30]; and salt bridges, <4Å [31]. Poor contact sites were defined as outside of reference distances or inconsistent with amino acid physicochemical properties. Groups with mostly poor contacts, those which included excessive backbone or intramolecular interactions or both, or appeared to be largely the result of non-specific shape fitting were not further analyzed. The remaining groups were evaluated based on the strength of the contact sites to identify a final group of poses as likely to represent the ligand-receptor complex. Contact sites on the ECLs were predicted when ligand residues docked at the top of the pocket and interactions just outside of reference ranges (less than 1Å) were considered if the contact could be made in a flexible *in vivo* system.

## Results

### CCK WMDF-NH_2_ docked to CCK-R1

**Figure 1:**
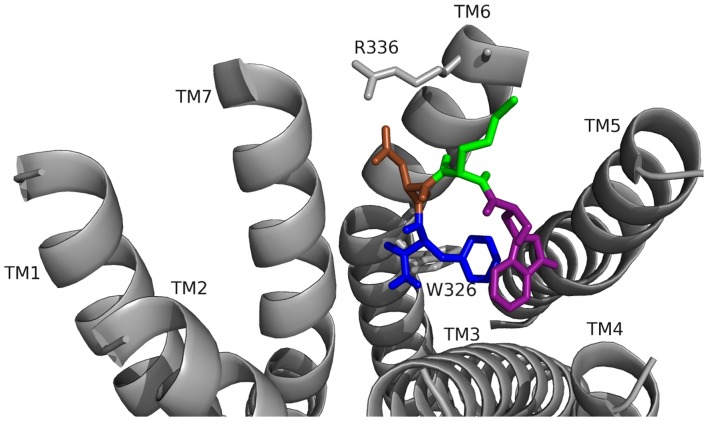
WMDF-NH_2_ docked to CCK-R1. Contacts between the amino acids inWMDF-NH_2_ are shown as W (purple), M (green), D (brown), and F (dark blue) and the receptor, FMRFa-R, whose transmembrane regions are labeled TM# where # is 1–7. The C-terminal F was amidated, -NH_2_.

To test the protocol used to identify FMRF-NH_2_ ligand-receptor interactions, WMDF-NH_2_, the C-terminal tetrapeptide of CCK peptides was docked to CCK-R1 and the results compared to published contact sites which were independently derived with different methodology [32]. WMDF-NH_2_ docking was analyzed blind, without consideration of published contact sites. In one of the three favorable poses of WMDF-NH_2_ docked to CCK-R1, the contacts for D and F matched published binding data (Figure 1, Table 1) [32]. D made a salt bridge with R336; F pi-stacked with W326 and was located in a hydrophobic pocket formed by residues on transmembrane 3 (TM3), TM4, TM5, TM6 and TM7; the exact experimentally-determined contact sites. This WMDF-NH_2_ pose showed the peptidyl backbone in a bent conformation due to pi-stacking between W and F which shared the hydrophobic pocket contact sites; the backbone shape resulted in M making hydrophobic contacts on TM6. In the N-terminal extended CCK peptide for which contact sites are published, the backbone extends across the receptor pocket and M makes contacts on TM1, TM2, and TM3 [32]; this conformation is probably stabilized by the additional N-terminal residues and their contact sites; however, the C-terminal tetrapeptide docking was assumed to contain representative contact sites. These findings validated the docking approach used in this study by predicting a pose which agrees with independent data.

**Table 1:** WMDF-NH_2_ ligand-receptor contact sites a.

W	Side chain	G122 ( 4.23Å)
		I172 (5.5Å)
		M173 (3.7Å)
		L213 (2.9Å)
		L214 (4.6Å)
		L217 (5.0Å)
		F4 (3.6–3.8Å)
	Backbone	—
M	Side chain	A337 (4.3Å)
	Backbone	N333 (O 3.0Å, H 3.5Å)
D	Side chain	R336 (3.1Å)
	Backbone	—
F	Side chain	G122 (4.7Å)
		V125 (4.6Å)
		L213 (4.6Å)
		L214 (4.4Å)
		W326 (3.6–3.9Å)
		I329 (4.7Å)
		F330 (5.1Å)
		W1 (3.6–3.8Å)
	Backbone	S359 (3.9Å)
NH_2_		S359 (4.1Å)

a One-letter amino acid codes followed by 1–4 are in the peptide; other numbers are in the receptor.

### FMRFa-R modeling

**Figure 2:**
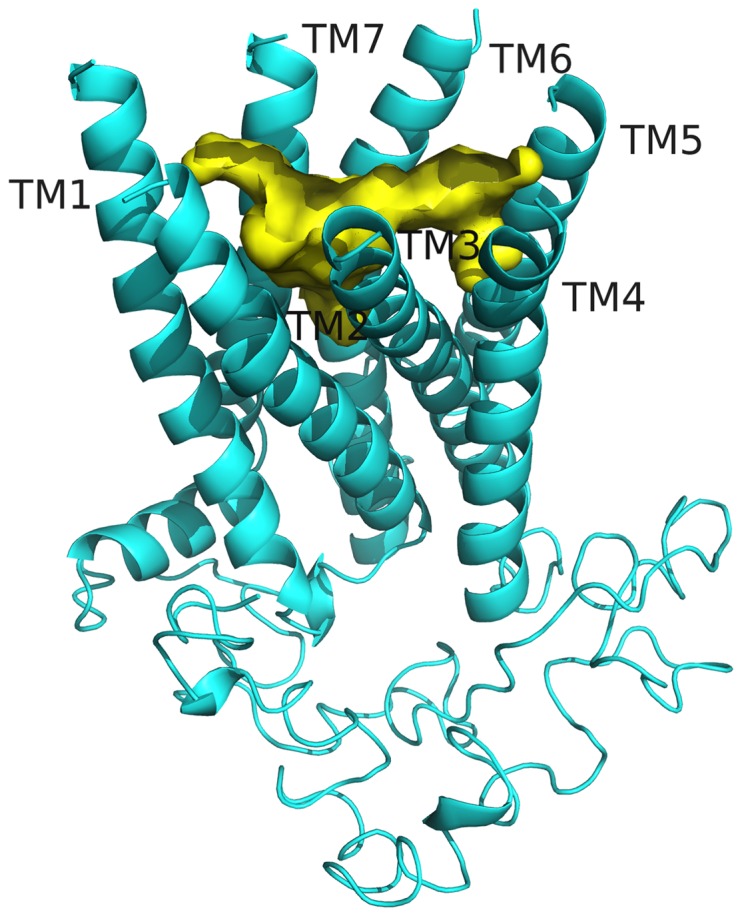
Model of the *D. melanogaster* FMRF-NH_2_ receptor. The receptor (cyan) is shown as a ribbon, with ECLs removed (top), retaining the seven TM helices (middle), the ICLs and intracellular tail (bottom). The predicted ligand binding pocket (yellow) stretches across the inside of the extracellular regions of the TM helices.

The model for FMRFa-R (Figure 2) contained features common to all GPCRs, including the 7 TM helix bundle, and extracellular and intracellular loops (ICLs) and tails. The model used in docking had no extracellular loops, as described previously [16], allowing the ligand to access the amino acid side chains exposed to the binding pocket. The region to which ligands were predicted to bind is formed by the extracellular regions of the TM helices; all ligand docking results were depicted showing this region. The ligand binding pocket stretches across the TMs, reaching several Ås deep into the region between the helices while also encompassing the area near the top of the pocket, where the predicted ECLs would exist.

### FMRF-NH_2_ docked to FMRFa-R

As a test of the hypothesis FMRF-NH_2_ was docked to FMRFa-R to identify contact sites, in particular, how F1 interacted with the receptor because its position defines the FaRP subgroup. Typical of rhodopsin-like GPCRs, FMRFa-R contained a 7 TM helix bundle which formed a putative ligand binding pocket [33]; peptides usually bind near the top of this pocket due to steric restrictions and make extensive contacts to the ECLs [34]; thus, side chains projecting into the pocket and on the ECLs are considered candidates for ligand-receptor contact sites.

**Figure 3:**
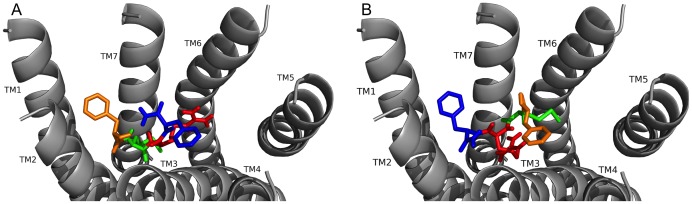
FMRF-NH_2_ docked to FMRFa-R yielded two best poses. Two best poses (A and B) were generated in docking FMRF-NH_2_ to FMRFa-R. Contacts made between the amino acids in FMRF-NH_2_ are shown as F1 (orange), M (green), R (red), and F4 (blue) and the receptor, FMRFa-R, whose transmembrane regions are labeled TM# where # is 1–7. The C-terminal F was amidated, -NH_2_.

When the conserved tetrapeptide FMRF-NH_2_ was docked two best poses were observed with equally favorable contact sites (Figures 3A and B, Tables 2 and 3). In both poses, the two F residues made multiple, strong pi-stacking and hydrophobic interactions, acting as anchors for peptide docking. M2 made hydrophobic interactions and R3 made hydrogen bonds; in both poses these residues were important as judged by their spanning the receptor pocket and making contacts to several TMs. Hydrogen bonds made by the C-terminal -NH_2_ and intramolecular hydrogen bonds within the peptide backbone also stabilized the docked conformations.

**Table 2:** FMRF-NH_2_ ligand-receptor contact sites, pose A a.

F	Side chain	F169 (3.7–3.8Å)
		P172 (3.7Å)
	Backbone	S165 (3.9Å)
		R3 (2.8Å)
		NH_2_ (3.7Å)
M	Side chain	L161 (3.5Å)
		I166 (4.6Å)
		G200 (3.8Å)
		I382 (4.0Å)
		T383 (3.6Å)
	Backbone	S165 (2.9Å)
R	Side chain	Q204 (2.3Å)
		Y301 (3.0Å)
		N379 (4.3Å)
	Backbone	F1 (2.8Å)
F	Side chain	P194 (3.4 Å)
		F197 (3.6–4.3Å)
	Backbone	—
NH_2_		S193 (3.9Å)
		F1 (3.7Å)

a One-letter amino acid codes followed by 1–4 are in the peptide; other numbers are in the receptor.

**Table 3:** FMRF-NH_2_ ligand-receptor contact sites, pose B a.

F	Side chain	P194 (3.7Å)
		F197 (3.7–4.0Å)
	Backbone	N379 (3.8Å)
M	Side chain	F194 (5.0Å)
		M201 (3.8Å)
		L358 (4.0Å)
		I362 (3.8Å)
	Backbone	R3 (3.5Å)
R	Side chain	S165 (2.3Å)
		Q204 (2.8Å)
		M2 (3.5Å)
	Backbone	F4 (2.7Å)
		NH_2_ (2.7)
F	Side chain	F169 (3.6–4.3Å)
		P172 (3.8 Å)
	Backbone	R3 (2.4Å)
NH_2_		S193 (3.8Å)
		R3 (2.7Å)

a One-letter amino acid codes followed by 1–4 are in the peptide; other numbers are in the receptor.

In pose A (Figure 3A, Table 2), the two F residues made pi-stacking and hydrophobic interactions; F1 docked near TM3 and F4 docked near TM1 and TM2. M2 made numerous hydrophobic contact sites deep in the pocket to form a network; the F1 and M2 backbones formed a small hydrogen-bonding network around S165 on TM2. R3 made hydrogen bonds to receptor residues on TM3 and TM5 to span the pocket and the C-terminal -NH_2_ was hydrogen bonded to S193 at the top of TM3. In pose B (Figure 3B, Table 3), F1 and F4 again made strong pi-stacking and hydrophobic contacts; however, F1 was docked near TM1 and TM2 and F4 was docked near TM3, the opposite of pose A. M2 formed several hydrophobic interactions on TM3 and TM5 to span the pocket. R3 was hydrogen bonded to S165 and Q204 on TM2 and TM3, and the backbone interacted with the C-terminal -NH_2_; the -NH_2_ made an additional hydrogen bond to S193, forming a small network.

### YMRF-NH_2_ docked to FMRFa-R

**Figure 4:**
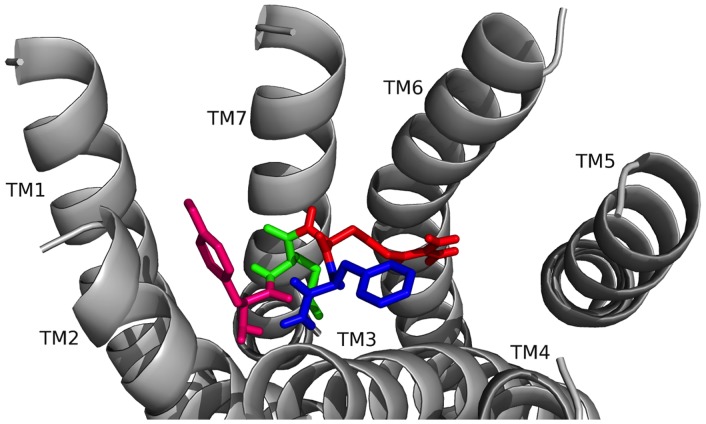
YMRF-NH_2_ docked to FMRFa-R. Contacts made between the amino acids in YMRF-NH_2_ are shown as Y (hot pink), M (green), R (red), and F4 (blue) and the receptor, FMRFa-R, whose transmembrane regions are labeled TM# where # is 1–7. The C-terminal F was amidated, -NH_2_.

Next, the analog YMRF-NH_2_, in which Y replaced F1, was docked to FMRFa-R (Figure 4) to identify contact sites, in particular, to investigate the importance of an aromatic residue in position 1 of the tetrapeptide. The YMRF-NH_2_ contact sites were nearly identical to FMRF-NH_2_ in pose A (Table 4). The only major difference in the contact sites for YMRF-NH_2_ compared to FMRF-NH_2_ was a hydrogen bond made by the added hydroxyl group of Y1. A second pose for YMRF-NH_2_ docked to FMRFa-R was not observed; this lack of a hydrogen bond contact site probably decreased the favorability for Y1 to dock near TM3 like F1 in FMRF-NH_2_ pose B.

**Table 4:** YMRF-NH_2_ ligand-receptor contact sites a.

Y	Side chain	F169 (3.5–4.4Å)
		P172 (3.7Å, OH 4.2Å)
	Backbone	S165 (2.2Å)
		NH_2_ (2.9Å)
M	Side chain	L161 (3.8Å)
		G200 (3.6Å)
		I382 (4.6Å)
		T383 (3.9Å)
	Backbone	—
R	Side chain	Q204 (2.5Å)
		Y301 (2.4Å)
	Backbone	N379 (3.4Å)
F	Side chain	P194 (3.5Å)
		F197 (3.6–3.9Å)
	Backbone	—
NH_2_		Y1 (2.9Å)

a One-letter amino acid codes followed by 1–4 are in the peptide; other numbers are in the receptor.

In the YMRF-NH_2_ docked pose (Figure 4, Table 4), the contact sites for the Y1, R3, and F4 side chains were identical to F1, R3, and F4 in FMRF-NH_2_ pose A; M2 was positioned slightly differently, but still made hydrophobic contact sites on several TMs. The Y1 side chain was rotated compared to F1 to accommodate a hydrogen bond made by the hydroxyl group. This rotation still allowed Y1 to make the same strong contacts as F1, especially a pi-stacking interaction with F169. Overall, YMRF-NH_2_ and pose A of FMRF-NH_2_ docked to FMRFa-R with a majority of the same contact sites and similar orientations in the pocket.

### AMRF-NH_2_ docked to FMRFa-R

**Figure 5:**
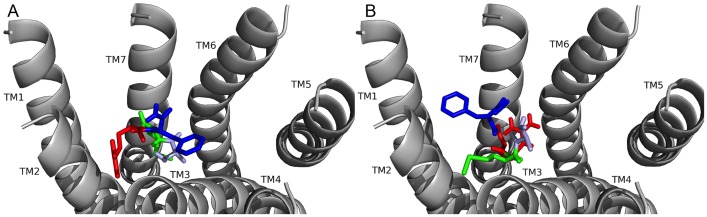
AMRF-NH_2_ docked to FMRFa-R yielded two best poses. Two best poses (A and B) were generated in docking AMRF-NH_2_ to FMRFa-R. Contacts made between the amino acids in AMRF-NH_2_ are shown as A (sky blue), M (green), R (red), and F4 (blue) and the receptor, FMRFa-R, whose transmembrane regions are labeled TM# where # is 1–7. The C-terminal F was amidated, -NH_2_.

FMRF-NH_2_ SAR was further explored by replacing F1 with an amino acid with a small, aliphatic side chain, A; AMRF-NH_2_ was docked to FMRFa-R. Similar to FMRF-NH_2_ docking, two poses existed for AMRF-NH_2_ docking with equally favorable contact sites (Figures 5A and B, Tables 5 and 6). The poses made some of the same contact sites observed for FMRF-NH_2_; however, because of the physicochemical differences between F and A, AMRF-NH_2_ lost strong pi-stacking contacts. As a result, AMRF-NH_2_ took on a different backbone shape, which altered contact sites compared to FMRF-NH_2_ and left AMRF-NH_2_ without any contact sites on TM4, TM5, or TM6 in either pose. Overall, these changes made the ligand-receptor interaction between AMRF-NH_2_ and FMRFa-R weaker than for FMRF-NH_2_.

**Table 5:** AMRF-NH_2_ ligand-receptor contact sites, pose A a.

A	Side chain	F197 (3.7Å)
		G200 (3.9Å)
		M2 (3.7Å)
	Backbone	—
M	Side chain	L161 (4.8Å)
		G200 (3.8Å)
		I382 (4.8Å)
		T383 (3.9Å)
		A1 (3.7Å)
	Backbone	N379 (3.7Å)
R	Side chain	S193 (3.2Å)
	Backbone	NH_2_ (2.7Å)
F	Side chain	P194 (3.7Å)
		F197 (3.7–3.9Å)
	Backbone	ECLs
NH_2_		ECLs
		R3 (2.7Å)

a One-letter amino acid codes followed by 1–4 are in the peptide; other numbers are in the receptor.

**Table 6:** AMRF-NH_2_ ligand-receptor contact sites, pose B a.

A	Side chain	P194 (4.4Å)
		F197 (3.9Å)
	Backbone	NH_2_ (2.8Å)
M	Side chain	L168 (5.0Å)
		P172 (4.2Å)
		I192 (4.2Å)
		V196 (4.4Å)
	Backbone	—
R	Side chain	Q204 (3.2Å)
		N379 (2.1Å)
	Backbone	—
F	Side chain	C116 (4.7Å)
		F169 (3.6–4.5Å)
		P172 (4.5Å)
	Backbone	N79 (2.1Å)
NH_2_		ECLs
		A1 (2.8Å)

a One-letter amino acid codes followed by 1–4 are in the peptide; other numbers are in the receptor.

In pose A (Figure 5A, Table 5), F4 docked near TM3 similar to pose A of FMRF-NH_2_ docking; M2 also initiated a hydrophobic network like M2 in pose A of FMRF-NH_2_. However, contact sites on TM1 observed for F1 of FMRF-NH_2_ were not made by AMRF-NH_2_ because the non-aromatic A1 could not pi-stack and was not large enough to make multiple interactions; A1 instead made hydrophobic contacts on TM3 not observed for F1 of FMRF-NH_2_. The new contact sites for A1 caused a change in the backbone shape which resulted in R3 making a hydrogen bond at the top of TM3 that was made by the C-terminal -NH_2_ in FMRF-NH_2_ pose A, and the C-terminal -NH_2_ being positioned for ECL contacts through an intramolecular hydrogen bond to R3.

In pose B (Figure 5B, Table 6), A1 docked near TM3 like F1 did in FMRF-NH_2_ pose B; however, A could not pi-stack because it has much less surface area than F does, thus, the contacts were more distanced and weaker. This caused a change in the backbone conformation of AMRF-NH_2_ compared to FMRF-NH_2_, which resulted in contact sites for M2 and R3 different from FMRF-NH_2_ pose B. R3 still contacted Q204 on TM3, but was positioned in the pocket differently and made a hydrogen bond on TM7 instead of the one on TM2 observed for FMRF-NH_2_. M2 made hydrophobic contacts on TM2 and TM3 not observed for FMRF-NH_2_, although F4 docked near TM1 to make the same contacts as in FMRF-NH_2_. The C-terminal -NH_2_ was positioned for ECL contacts by an intramolecular hydrogen bond to the A1 backbone.

### FMRF-NH_2_ and analogs docked to FMRFa-R: Conclusions

This set of docking experiments showed the conserved tetrapeptide FMRF-NH_2_ made numerous, strong contacts (Figure 3, Tables 2 and 3) throughout the FMRFa-R binding pocket which likely allowed it to bind to and activate the receptor, consistent with FMRF-NH_2_ binding to expressed FMRFa-R protein [10] and its cardioexcitatory effect [1]. However, the C-terminal tetrapeptide activity remained untested compared to the five naturally-occurring FMRFa-extended peptides in a *D. melanogaster* bioassay. In addition, the results tested and supported the hypothesis that F1 made extensive and strong ligand-receptor contacts. Replacement of F1 with Y illustrated how an aromatic residue could substitute for the amino acid which defines position 1 of this subgroup of RF-NH_2_-containing peptides, yet a small, aliphatic residue, A, cannot.

The docking data were consistent with ligand-FMRFa-R binding with the reported EC_50_ values of 28 nM (FMRF-NH_2_), 31 nM (YMRF-NH_2_) and 3,217 nM (AMRF-NH_2_) [10]; the nearly identical contact sites of FMRF-NH_2_ pose A and YMRF-NH_2_ (Figures 3A and 4, Tables 2 and 4) are consistent with the two peptides binding to FMRFa-R with a similar EC_50_. In contrast, when the small, non-aromatic residue A was substituted for F, strong contacts which anchored FMRF-NH_2_ in the FMRFa-R binding pocket were lost, which substantially weakened ligand-receptor interactions (Figure 5, Tables 5 and 6). In addition, the F1→A analog also took on new conformations, which resulted in neither of the AMRF-NH_2_ poses making contacts on TM4, TM5, or TM6; interacting with only half of the receptor pocket is expected to make AMRF-NH_2_ binding much weaker and less favorable than FMRF-NH_2_ binding. These results were consistent with AMRF-NH_2_ binding to expressed FMRFa-R protein at a higher EC_50_ (3,217 nM) [10]. Together, the binding and docking data for FMRF-NH_2_, YMRF-NH_2_, and AMRF-NH_2_ illustrated the importance of the aromatic residues in anchoring the peptide and maintaining docked conformations with favorable contact sites.

### PDNFMRF-NH_2_ and SDNFMRF-NH_2_ docked to FMRFa-R

**Figure 6:**
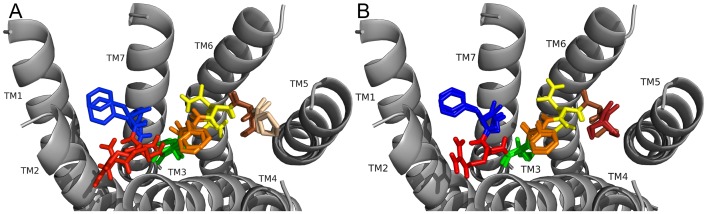
PDNFMRF-NH_2_ and SDNFMRF-NH_2_ docked to FMRFa-F. PDNFMRF-NH_2_ docked to FMRFa-R (see A). Contacts made between the amino acids in PDNFMRF-NH_2_ are shown as P (tan), D (brown), N (yellow), F4 (orange), M (green), R (red), and F7 (blue) and the receptor, FMRFa-R, whose transmembrane regions are labeled TM# where # is 1–7. The C-terminal F was amidated, -NH_2_. SDNFMRF-NH_2_ docked to FMRFa-R (see B). Contacts made between the amino acids in SDNFMRF-NH_2_ are shown as S (brick red), D (brown), N (yellow), F4 (orange), M (green), R (red), and F7 (blue) and the receptor, FMRFa-R, whose transmembrane regions are labeled TM# where # is 1–7. The C-terminal F was amidated, -NH_2_.

The next experiments explored how the unique N-terminal extensions influenced docking. The poses generated for PDNFMRF-NH_2_ and SDNFMRF-NH_2_ docking to FMRFa-R were viewed (Figures 6A and B). As predicted based on the structural similarity of the ligands, PDNFMRF-NH_2_ and SDNFMRF-NH_2_ docked to FMRFa-R with nearly identical contact sites (Tables 7 and 8); the only difference was for the N-terminal residue, which is consistent with differences in their physicochemical properties. In general, the two peptides docked in the FMRFa-R binding pocket with their N termini near TM4 and TM5 and their identical C-terminal tetrapeptide near TM1 and TM2. There were two equally favorable poses for PDNFMRF-NH_2_ and SDNFMRF-NH_2_ that were identical except for the position of N3. There was no evidence for one of the two positions of N3 to be more favorable than the other. Based on the number, strength, and diversity of contact sites, the F4 and F7 in PDNFMRF-NH_2_ and SDNFMRF-NH_2_ interactions with FMRFa-R appeared the strongest and acted as anchors for peptide docking, similar to how the two F residues, F1 and F4, acted in FMRF-NH_2_ docking.

**Table 7:** PDNFMRF-NH_2_ ligand-receptor contact sites a.

P	Side chain	V259 (3.6Å)
		I292 (4.2Å)
		Y296 (4.5Å)
		ECLs
	Backbone	R255 (3.1Å)
		H293(4.1Å)
D	Side chain	R255 (2.4Å, 3.3Å)
		N3 (2.4Å, 3.3Å)
	Backbone	N3 (2.1Å)
N	Side chain	Q204 (O 3.1Å, H 2.9Å)
		Y301 (2.9 Å)
		N379 (O 4.1Å, H 2.3Å)
		D2 (2.1Å)
		N3 (2.4Å)
	Backbone	D2 (2.4Å, 3.3Å)
		N3 (2.4Å)
F	Side chain	P194 (3.8Å)
		F197 (3.4–4.0Å)
	Backbone	—
M	Side chain	L161 (4.5Å)
		G200 (3.7Å)
		I382 (4.2Å)
		T383 (3.8Å)
	Backbone	S165 (3.6 Å)
R	Side chain	S193 (2.2Å)
		ECL1
	Backbone	—
F	Side chain	C116 (4.3Å)
		F169 (3.9–4.5Å)
		P172 (3.7Å)
		L380 (5.0Å)
	Backbone	N379 (3.3Å)
NH_2_		N379 (3.8Å)

a One-letter amino acid codes followed by 1–7 are in the peptide; other numbers are in the receptor.

**Table 8:** SDNFMRF-NH_2_ ligand-receptor contact sites a.

S	Side chain	R255 (2.3Å)
		ECLs
		D2 (2.4Å)
	Backbone	R255 (3.4Å)
D	Side chain	R255 (3.1Å, 3.5Å)
		S1 (2.4Å)
		N3 (2.8Å)
	Backbone	N3 (3.1Å)
N	Side chain	Q204 (O 2.4Å, H 3.8Å)
		Y301 (3.6Å)
		N379 (O 3.1Å, H 3.9Å)
		D2 (2.8Å)
		N3 (2.4Å)
	Backbone	D2 (2.8Å)
		N3 (2.4Å)
F	Side chain	P194 (4.3 Å)
		F197 (3.5–4.1Å)
	Backbone	NH_2_ (3.5Å)
M	Side chain	L161 (4.3Å)
		G200 (3.8Å)
		I382 (4.4Å)
		T383 (4.8Å)
	Backbone	S165 (3.5Å)
R	Side chain	S193 (3.1Å)
		ECL1
	Backbone	—
F	Side chain	C116 (4.3Å)
		F169 (3.6–4.7Å)
		P172 (4.7Å)
		L380 (5.0Å)
	Backbone	N379 (2.9Å)
NH_2_		N379 (3.8Å)
		F4 (3.5Å)

a One-letter amino acid codes followed by 1–7 are in the peptide; other numbers are in the receptor.

In the docked pose for PDNFMRF-NH_2_ (Figure 6A, Table 7), P1 made hydrophobic contacts on TM4 and TM5, and its backbone was hydrogen bonded to R255 and H293. D2 formed a salt bridge with R255 on TM4. In one position N3 had hydrogen bonds to N379 on TM6 and its own backbone, and in the other N3 was hydrogen bonded to Q204 on TM3 along with the D2 backbone or side chain. F4 pi-stacked with F197 and made a hydrophobic contact to P194 on TM3. M5 made hydrophobic contacts on several TMs deep in the pocket to form a network. R6 had a hydrogen bond to S193 on TM3, which positioned it to make additional contacts on ECL1; it would likely cation-pi bond with one of several aromatic residues located there. F7 pi-stacked with F169 on TM1 and made additional hydrophobic contacts, its backbone hydrogen bonded to N379. The C-terminal -NH_2_ also hydrogen bonded to N379. The ligand-receptor contacts for SDNFMRF-NH_2_ (Figure 6B, Table 8) were identical to PDNFMRF-NH_2_ except for the N-terminal residue; S hydrogen bonded to R255 and D2 instead of making the hydrophobic contacts observed for P.

### DPKQDFMRF-NH_2_ and SPKQDFMRF-NH_2_ docked to FMRFa-R

**Figure 7:**
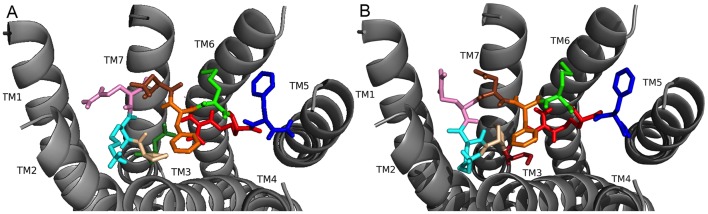
DPKQDFMRF-NH_2_ and SPDKQDFMRF-NH_2_ docked to FMRFa-R. Contacts made between the amino acids in DPKQDFMRF-NH_2_ are shown as D (forest green), P (tan), K (cyan), Q (light pink), D (brown), F6 (orange), M (green), R (red), and the C-terminal F as dark blue and the receptor, FMRFa-R, whose transmembrane regions are labeled TM# where # is 1–7 (see A). The C-terminal F was amidated, -NH_2_. Contacts made between the amino acids in SPKQDFMRF-NH_2_ are shown as S (brick red), P (tan), K (cyan), Q (light pink), D (brown), F6 (orange), M (green), R (red), and F9 (blue) and the receptor, FMRFa-R, whose transmembrane regions are labeled TM# where # is 1–7 (see B). The C-terminal F was amidated, -NH_2_.

In order to continue analysis of the role the unique N-terminal extensions played in ligand-receptor interactions DPKQDFMRF-NH_2_ and SPKQDFMRF-NH_2_ were docked to FMRFa-R (Figures 7A and B), and the contact sites observed are summarized in Tables 9 and 10. As predicted based on the structural similarity of the ligands_,_ the contact sites were very similar for the two peptides; the C termini docked near TM5 and TM6 and the N-termini docked near TM2 and TM3. Notably, contact sites for F6 in DPKQDFMRF-NH_2_ and SPKQDFMRF-NH_2_ were identical to those of F4 in PDNFMRF-NH_2_ and SDNFMRF-NH_2_ docking, and K3 made similar contacts to R6 in PDNFMRF-NH_2_ and SDNFMRF-NH_2_. However, the rest of the contact sites and orientation in the binding pocket were very different. The number, strength, and diversity of contact sites made the conserved residues F6 and F9 anchors for docking similar to the other peptides; multiple, close-range interactions with both the receptor and other ligand residues suggested R8 was also an anchor for DPKQDFMRF-NH_2_ and SPKQDFMRF-NH_2_ docking. A major difference between the DPKQDFMRF-NH_2_ and SPKQDFMRF-NH_2_ poses was in the position of P2; due to size differences between D1 and S1, P2 was positioned differently to allow D and S to make similar contacts.

**Table 9:** DPKQDFMRF-NH_2_ ligand-receptor contact sites a.

D	Side chain	Q204 (3.0Å)
		R8 (2.3Å)
	Backbone	S165 (3.6Å)
P	Side chain	V196 (4.2Å)
		G200 (4.9Å)
	Backbone	—
K	Side chain	S193 (2.3Å)
		ECL1
	Backbone	D5 (2.8Å)
Q	Side chain	P172 (3.9Å)
		ECLs
	Backbone	N379 (2.9Å)
		D5 (2.9Å)
D	Side chain	ECLs
		K3 (2.8Å)
		Q4 (2.9Å)
		F6 (2.5Å)
	Backbone	M7 (3.2Å)
F	Side chain	P194 (4.2Å)
		F197 (3.6–4.4Å)
	Backbone	D5 (2.5Å)
		R8 (2.7Å)
M	Side chain	L358 (4.3Å)
		I362 (5.0Å)
		A365 (4.0Å)
		T375 (3.7Å)
		F9 (3.7Å)
	Backbone	D5 (3.2Å)
		F9 (2.2Å)
R	Side chain	Q204 (2.5Å)
		N379 (2.5Å)
		D1 (2.3Å)
		F6 (2.7Å)
	Backbone	—
F	Side chain	H293 (3.5–4.5Å)
		I362 (4.5Å)
		F366 (3.2Å)
		ECLs
		M7 (3.7Å)
	Backbone	R255 (3.1Å)
		M7 (2.2Å)
NH_2_		N289 (2.4Å)
		ECLs

a One-letter amino acid codes followed by 1–9 are in the peptide; other numbers are in the receptor.

**Table 10:** SPKQDFMRF-NH_2_ ligand-receptor contact sites a.

S	Side chain	Q204 (3.8Å)
		R8 (2.8Å)
	Backbone	S165 (2.2Å)
P	Side chain	F169 (3.7Å)
		T383 (4.2Å)
	Backbone	S165 (3.6Å)
K	Side chain	S193 (2.4Å)
		ECL1
	Backbone	D5 (3.4Å)
Q	Side chain	D5 (2.2Å)
		ECLs
	Backbone	N379 (2.9Å)
		D5 (3.1Å)
D	Side chain	ECLs
		Q4 (2.2Å)
	Backbone	K3(3.4Å)
		Q4 (3.1Å)
F	Side chain	P194 (5.1Å)
		F197 (3.9–4.4Å)
	Backbone	N379 (3.7Å)
		R8 (2.1Å)
M	Side chain	L358 (4.0Å)
		I362 (3.9Å)
		A365 (3.9Å)
		T375 (4.0Å)
	Backbone	F9 (2.7Å)
R	Side chain	Q204 (2.6Å)
		N379 (2.2Å)
		S1 (2.8Å)
		F6 (2.1Å)
	Backbone	—
F	Side chain	H293 (3.6–4.6Å)
		I362 (4.5Å)
		F366 (3.3Å)
		ECLs
	Backbone	R255 (3.1Å)
		M7 (2.7Å)
NH_2_		N289 (5.3Å)
		ECLs

a One-letter amino acid codes followed by 1–9 are in the peptide; other numbers are in the receptor.

For DPKQDFMRF-NH_2_ docking (Figure 7A, Table 9), D1 was hydrogen bonded to Q204 on TM3 and made an intramolecular salt bridge with R8; its backbone was hydrogen bonded to S165 on TM2. The P2 ring structure contacted hydrophobic residues on TM2 and its turn-inducing properties were critical for positioning D1 to make its contacts. K3 hydrogen bonded to S193 on TM2 which positioned it for contacts with aromatic residues on ECL1. Q4 hydrogen bonded to exposed backbones at the top of TM2 to position it for ECL contacts and its backbone hydrogen bonded to N379 on TM7. D5 formed hydrogen bonds with the backbones of K3, Q4, and F6 to stabilize the docked conformation and was also positioned for ECL contacts. F6 pi-stacked with F197 and had a hydrophobic contact with P194 on TM3. M7 made several hydrophobic contacts on TM6 and TM7; with an intramolecular contact to F9 it formed a hydrophobic network. R8 was hydrogen bonded to Q204, N379, and the F6 backbone in addition to the conformation-stabilizing salt bridge to D1, these contacts formed a network of hydrophilic interactions. F9 pi-stacked with H293 and made several other hydrophobic interactions, its backbone hydrogen bonded to the M7 backbone and R255 on TM4. The C-terminal -NH_2_ hydrogen bonded to N289 and several intramolecular hydrogen bonds between backbones stabilized the docked conformation.

For SPKQDFMRF-NH_2_, the C-terminal FMRF-NH_2_ docked identically to DPKQDFMRF-NH_2_ and its full length backbone had a similar overall orientation in the pocket (Figures 7A and B). However, there were notable differences which would be anticipated because of the difference in size between S and D (Figures 7A and B, Tables 9 and 10). First, P2 was no longer oriented near TM2, but was positioned towards the middle of the pocket to make hydrophobic contacts on TM2 and TM7. This position allowed S1 to make its contacts, which were similar to D1 in DPKQDFMRF-NH_2_ but with a hydrogen bond to R8 instead of a salt bridge. Because of the different P2 position, the backbone shape was altered slightly, causing the D5 and Q4 side chains to hydrogen bond, a contact not observed for DPKQDFMRF-NH_2_; both side chains maintained contacts on the ECLs and structure-stabilizing backbone interactions observed for DPKQDFMRF-NH_2_. The K3 backbone also did not form an intramolecular hydrogen bond with the D5 side chain as observed for DPKQDFMRF-NH_2_, an additional consequence of the altered backbone shape.

### TPAEDFMRF-NH_2_ docked to FMRFa-R

**Figure 8:**
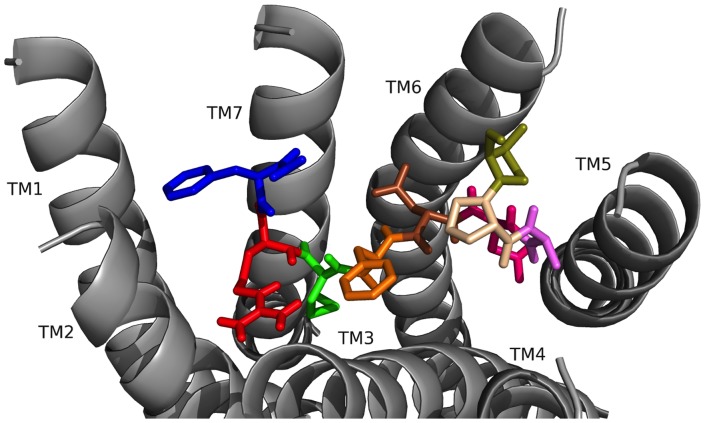
TPAEDFMRF-NH_2_ docked to FMRFa-R. Contacts made between the amino acids in TPAEDFMRF-NH_2_ are shown as T (olive green), P (tan), A (lavender), E (hot pink), D (brown), F6 (orange), M (green), R (red), and F9 (blue) and the receptor, FMRFa-R, whose transmembrane regions are labeled TM# where # is 1–7. The C-terminal F was amidated, -NH_2_.

Next, the docking results for TPAEDFMRF-NH_2_ were analyzed to further gain insight into whether the N-terminal extension influenced ligand-receptor interactions. The TPAEDFMRF-NH_2_ N terminus oriented in the FMRFa-R binding pocket near TM4 and TM5 with the C terminus near TM1 and TM2 (Figure 8). TPAEDFMRF-NH_2_ made contacts (Table 11) to TM3, TM4, TM5, and TM6; in particular, the flexible A and turn-inducing P facilitated a bend in the peptidyl backbone which allowed T1 to make contacts with residues on TM5, TM6, and the ECLs. The two F residues in the C-terminal FMRF-NH_2_ again acted as anchors for docking. F1 of the conserved C terminus made identical contact sites on TM3 to the other four extended FMRF-NH_2_ peptides.

**Table 11:** TPAEDFMRF-NH_2_ ligand-receptor contact sites a.

T	Side chain	N289 (4.6Å, 4.9Å)
		H293 (4.1Å)
		F366 (3.8Å)
		ECLs
		E4 (2.1Å)
	Backbone	A3 (1.9Å)
		E4 (3.3Å)
P	Side chain	F197 (5.1Å)
		ECLs
		F6 (3.9Å)
	Backbone	R255 (3.8Å)
A	Side chain	V259 (4.1Å)
		I292 (4.2Å)
		Y296 (3.8Å)
	Backbone	T1 (1.9Å)
E	Side chain	R255 (3.1Å)
		Y301 (4.0Å)
	Backbone	T1 (2.1Å)
D	Side chain	N379 (3.3Å)
		F6 (2.2Å)
	Backbone	—
F	Side chain	P194 (4.3Å)
		F197 (3.8–4.0Å)
	Backbone	D5 (2.2Å)
M	Side chain	V196 (4.9Å)
		G200 (4.0Å)
		Q204 (3.9Å)
		I382 (4.4Å)
		T383 (4.1Å)
	Backbone	—
R	Side chain	S193 (2.6Å)
		ECL1
	Backbone	—
F	Side chain	C116 (4.1Å)
		L161 (3.9Å)
		F169 (3.6–6.3Å)
		P172 (5.0Å)
		L380 (4.8Å)
	Backbone	N379 (3.0Å)
NH_2_		ECLs

a One-letter amino acid codes followed by 1–9 are in the peptide; other numbers are in the receptor.

The T1 hydroxyl in TPAEDFMRF-NH_2_ (Figure 8, Table 11) made hydrogen bonds to H293, N289, and the E4 backbone, and the T1 methyl group made a hydrophobic contact to F366; these contacts positioned T1 for additional interactions with the ECLs. The T1 backbone hydrogen bonded to the A3 and E4 backbones to stabilize the docked conformation. P2 made hydrophobic contacts to F197 on TM3 and F6; the P2 backbone also hydrogen bonded to R255. A3 made hydrophobic contacts to V259, I292, and Y296, forming a small hydrophobic network. E4 made a salt bridge to R255 and was also hydrogen bonded to Y301. D5 hydrogen bonded to N379 and the F6 backbone; the F6 side chain pi-stacked with F197 and made a hydrophobic contact to P194. M7 formed a network of hydrophobic interactions deep in the pocket, as well as a hydrogen bond between its sulfur atom and Q204. R8 was hydrogen bonded to S193 at the top of TM3 which positioned it for contacts on ECL1; it would likely cation-pi bond with one of several aromatic residues located there. F9 pi-stacked with F169 and made hydrophobic contacts on TM1 and TM7, and the F9 backbone was hydrogen bonded to N379. The C-terminal -NH_2_ did not contact the TMs of FMRFa-R, although it was positioned to make ECL contacts.

### FMRF-NH_2_ peptides docked to FMRFa-R: Conclusions

These docking results illustrated the N-terminal residues of the five *D. melanogaster* FMRF-NH_2_-containing peptides played roles in ligand-receptor docking, thus, supporting the hypothesis driving this study. This conclusion was supported by PDNFMRF-NH_2_ and SDNFMRF-NH_2_ taking on markedly different orientations from DPKQDFMRF-NH_2_ and SPKQDFMRF-NH_2_ in the FMRFa-R binding pocket (Figures 6 and 7, Tables 7–10). Although TPAEDFMRF-NH_2_ occupied the pocket similarly to PDNFMRF-NH_2_ and SDNFMRF-NH_2_, it had unique contacts not made by the other ligands and lacked extensive contacts with TM7; this was a result of its distinct N-terminal structure (Figures 6, 7, and 8, Tables 7–11). None of the peptides made contact sites for the C-terminal FMRF-NH_2_ identical to those for the tetrapeptide docked alone (Figure 3, Tables 2 and 3); in fact, none of the 200 results for the C-terminal FMRF-NH_2_ docking made similar contact sites to the tetrapeptide alone in DPKQDFMRF-NH_2_ and SPKQDFMRF-NH_2_ with the C-terminal F docked between TM5 and TM6. These results are consistent with the N terminus influencing docking.

Similar to the FMRF-NH_2_ docking results, the two F residues were important for all five peptides as anchors for ligand-receptor interactions. This was consistent with their strict conservation and the conclusion that AMRF-NH_2_ does not bind FMRFa-R because the small, non-aromatic A cannot make the same strong contacts as an F (Figure 5, Tables 5 and 6). In addition, there were several contact sites made by all five FMRF-NH_2_-containing peptides and FMRF-NH_2_ which may be linked to their similar activities. All six ligands made pi-stacking or hydrophobic contacts to F197, a hydrogen bond to S193, a hydrogen bond to Q204, and one or more hydrogen bonds to N379. The combination of these four contact points may be required for peptide binding and/or activity at FMRFa-R.

### FMRF-NH_2_ peptides applied to heart

**Figure 9:**
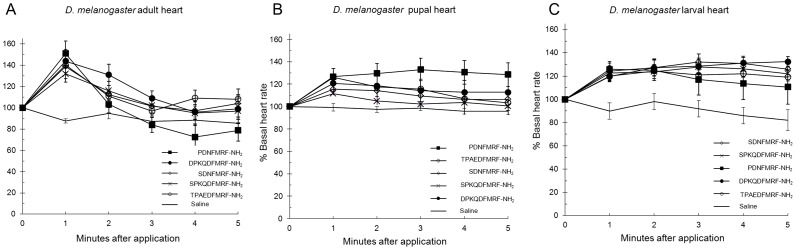
FMRF-NH_2_-containing peptides applied to heart. The effects of the five FMRF-NH_2_-containing peptides on heart rate are reported as mean values ± S.E.M. The effects are shown in adult (A), pupal (B), and larval (C) heart.

To continue SAR analysis, the five *D. melanogaster* FMRF-NH_2_ peptides were tested in a bioassay; the peptides were applied to adult, larval, and pupal heart at 1 µM. Although previous studies analyzed the effect of some or all of the peptides encoded in FMRFa in bioassays [35]–[40], none were conducted in multiple developmental stages nor were they compared to SAR, docking, or binding data. In all three stages, saline, the carrier, had no substantial effect on heart rate; statistical significance of peptide activity was measured compared to saline with p value < 0.01 considered significant. On adult heart, the FMRF-NH_2_-containing peptides increased heart rate compared to baseline; the effects were significantly different from saline (Figure 9A, Table 12). PDNFMRF-NH_2_ increased heart rate by 51±12%, SDNFMRF-NH_2_ by 39±11%, DPKQDFMRF-NH_2_ by 44±11%, SPKQDFMRF-NH_2_ by 32±8%, and TPAEDFMRF-NH_2_ by 40±2% (Table 12). All five peptides elicited maximum effects within 1 minute after application and heart rate returned to baseline within the 5 minute recording period (Figure 9A). Each of the peptides was cardioexcitatory: however, subtleties in the magnitude of the effects and the time courses of the responses existed.

**Table 12:** Cardioexcitatory effects of *D. melanogaster* FMRF-NH_2_ peptides and FMRF-NH_2_ a, b.

		% increase in heart rate					
		Adult	p value	Pupal	p value	Larval	p value
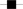	PDNFMRF-NH_2_	51±12	0.0006	27±7	0.004	26±5	0.003
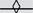	SDNFMRF-NH_2_	39±11	0.002	15±4	0.009	24±8	0.002
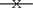	DPKQDFMRF-NH_2_	44±11	0.001	21±5	0.006	20±5	0.006
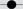	SPKQDFMRF-NH_2_	32±8	0.0002	12±1	0.002	23±6	0.005
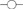	TPAEDFMRF-NH_2_	40±2	0.00002	26±8	0.004	20±4	0.004
	FMRF-NH_2_	37±11	0.003	17±4	0.003	28±8	0.005
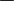	Saline	–13±2		1±3		–10±7	

a The effects of the five FMRF-NH_2_ peptides on adult, pupal, and larval heart are reported as mean values ± S.E.M. calculated as percent increase in heart rate with p values relative to saline.

b Basal heart rate is equal to 100%.

On pupal heart, all five of the *D. melanogaster* FMRF-NH_2_-containing peptides were also cardioexcitatory and increased heart rate compared to baseline with activities significantly different from saline (Figure 9B, Table 12). However, the magnitudes of these effects were smaller and the time courses varied compared to adult heart; PDNFMRF-NH_2_ increased heart rate by 27±7%, SDNFMRF-NH_2_ by 15±4%, DPKQDFMRF-NH_2_ by 21±5%, SPKQDFMRF-NH_2_ by 12±1%, and TPAEDFMRF-NH_2_ by 26±8% (Table 12). For four of the peptides, maximum effects occurred within 1 minute of application and heart rate returned to baseline within the 5 minute recording period; however, after application of PDNFMRF-NH_2_ heart rate did not return to baseline within the recording period (Figure 9B).

On larval heart, all five of the *D. melanogaster* FMRF-NH_2_-containing peptides increased heart rate compared to baseline and were significantly different from saline (Figure 9C, Table 12). The magnitudes of these effects and the time courses varied compared to adult heart; PDNFMRF-NH_2_ increased heart rate by 26±5%, SDNFMRF-NH_2_ by 24±8%, DPKQDFMRF-NH_2_ by 20±5%, SPKQDFMRF-NH_2_ by 23±6%, and TPAEDFMRF-NH_2_ by 20±4% (Table 12).

### FMRF-NH_2_ applied to heart

**Figure 10:**
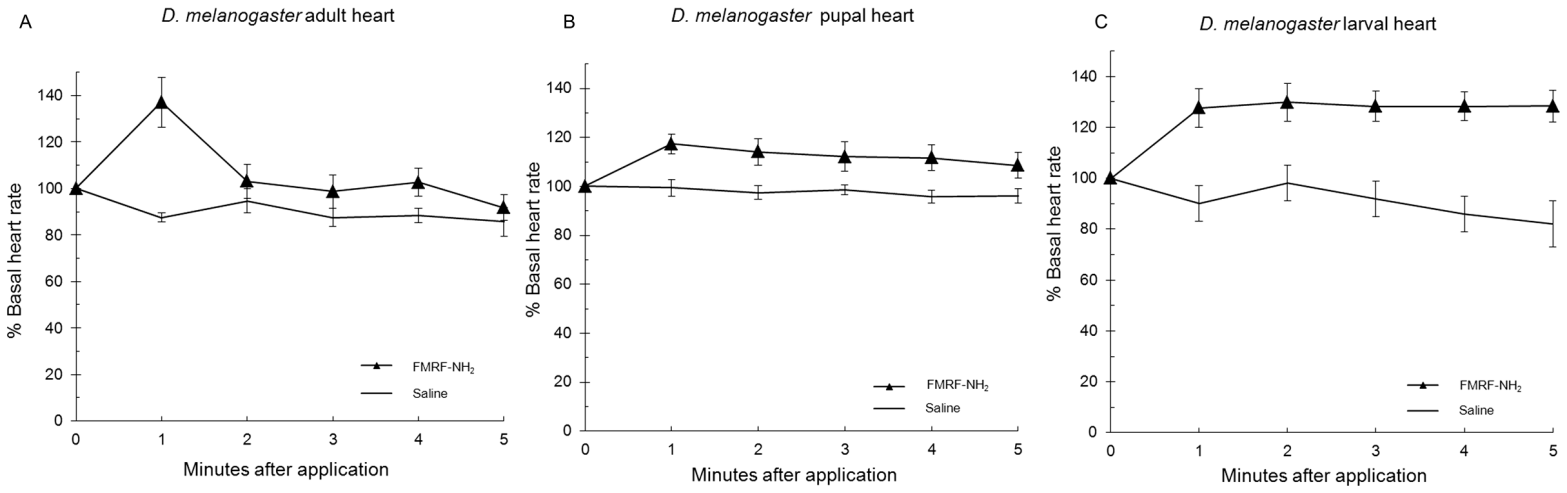
FMRF-NH_2_ applied to heart. The effect of the conserved tetrapeptide FMRF-NH_2_ on heart rate is reported as mean values ± S.E.M. The effects are shown in adult (A), pupal (B), and larval (C) heart.

Next, the effects of FMRF-NH_2_ were measured on heart rate at 1 µM; the C-terminal tetrapeptide binds to FMRFa-R protein [10]. In adult, pupa, and larva FMRF-NH_2_ increased heart rate by 37±11%, 17±4%, and 28±8%, effects significantly different from saline (Figure 10, Table 12). This is consistent with the ability of the tetrapeptide to bind FMRFa-R [10] and activate the receptor similarly to the N-terminally extended peptides.

### FMRF-NH_2_ peptides and FMRF-NH_2_ applied to heart: conclusions

The bioassay data demonstrated all five FaRPs elicited cardioactive responses in each of the three developmental stages tested; the tetrapeptide FMRF-NH_2_ was also cardioexcitatory in larva, pupa, and adult (Figures 9 and 10). These results were consistent with the importance of the strictly conserved C terminus in making contacts with FMRFa-R to bind to the receptor and activate the signaling pathway. Yet, the N-terminal extensions of the naturally-occurring peptides demonstrated unique contact sites; whether these differences were reflected in the results of the bioassay used may come from a further examination of the data. The magnitudes of the increases in heart rate were not the same in the three developmental stages; adult was the most robust, pupal and larval hearts were less responsive. Additionally, the effects over time varied in larva, pupa, and adult. These results may reflect the influence of the N-terminal extensions and the different peptide-receptor interactions.

### SDNAMRF-NH_2_ docked to FMRFa-R

**Figure 11:**
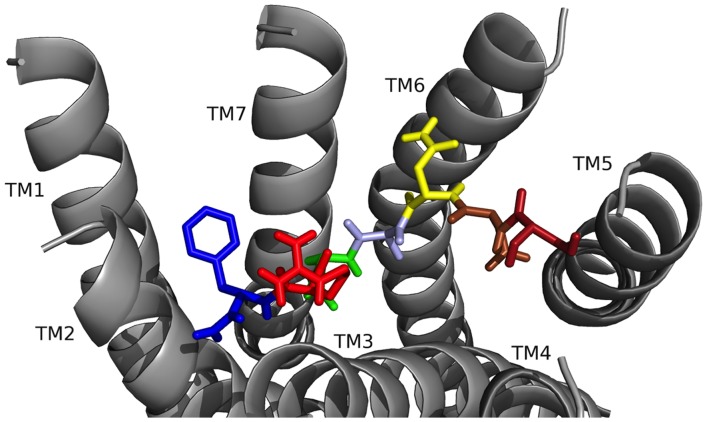
SDNAMRF-NH_2_ docked to FMRFa-R. Contacts between the amino acids in SDNAMRF-NH_2_ are shown as S (brick red), D (tan), N (yellow), A (sky blue), M (green), R (red), and F7 (blue) and the receptor, FMRFa-R, whose transmembrane regions are labeled TM# where # is 1–7. The C-terminal F was amidated, -NH_2_.

Based on the SAR data demonstrating F1 was important to anchor the tetrapeptide to the receptor and in binding to FMRFa-R, the analog SDNAMRF-NH_2_ was designed to test whether the N-terminal extension influenced docking and activity. SDNAMRF-NH_2_ docked to FMRFa-R with a similar orientation in the pocket to SDNFMRF-NH_2_ (Figure 11). Overall, a majority of the ligand-receptor contact sites were the same, yet, because of the F to A substitution, there were some slight changes (Table 13). The small, aliphatic amino acid A was not able to compensate for F, an aromatic residue; thus, it did not make the same contacts, which caused changes in the backbone shape of SDNAMRF-NH_2_ compared to SDNFMRF-NH_2_. As a result of the new backbone shape, there was only one possible conformation for N3, R6 made its contacts from a different angle, and the C-terminal –NH_2_ had no contacts. F7 remained an anchor for docking with strong, aromatic contact sites.

**Table 13:** SDNAMRF-NH_2_ ligand-receptor contact sites a.

S	Side chain	N289 (2.5Å)
		ECLs
	Backbone	R255 (4.0Å)
D	Side chain	R255 (3.2Å)
	Backbone	R255 (3.5Å)
N	Side chain	N379 (4.1Å)
		ECLs
	Backbone	—
A	Side chain	F197 (5.3Å)
		M201 (3.9Å)
		L358 (4.0Å)
	Backbone	N379 (3.4Å)
M	Side chain	L161 (4.0Å)
		G200 (3.9Å)
		L382 (4.0Å)
		T383 (3.6Å)
	Backbone	Q204 (3.4Å)
R	Side chain	S193 (2.8Å)
		ECL1
		F7 (3.0Å)
	Backbone	—
F	Side chain	F169 (3.5–3.8Å)
		P172 (3.5Å)
	Backbone	S165 (4.2Å)
		R6 (3.0Å)
NH_2_		—

a One-letter amino acid codes followed by 1–4 are in the peptide; other numbers are in the receptor.

In the SDNAMRF-NH_2_ docked pose (Figure 11, Table 13), S1, D2, M5, and F7 made the same contacts as they did in SDNFMRF-NH_2_. A4 made a distanced hydrophobic contact to F197 along with two hydrophobic contacts nearby and a hydrogen bond between its backbone and N379, contacts which were markedly different from F4 in SDNFMRF-NH_2_. The new contacts for A4 and the increased flexibility of the peptide resulted in a slightly different backbone conformation for SDNAMRF-NH_2_ compared to SDNFMRF-NH_2_; this caused slight changes in the contact sites of the remaining residues and a new hydrogen bond between the M5 backbone and Q204. Unlike SDNFMRF-NH_2_, there was only one orientation observed for N3; it maintained hydrogen bonds to N379, although at a different angle. R6 maintained a hydrogen bond to S193 which positioned it to interact with residues on ECL1, although also from a slightly different angle. The C-terminal -NH_2_ had no contacts, a change that resulted from the new backbone conformation.

### SDNAMRF-NH_2_ applied to heart

**Figure 12:**
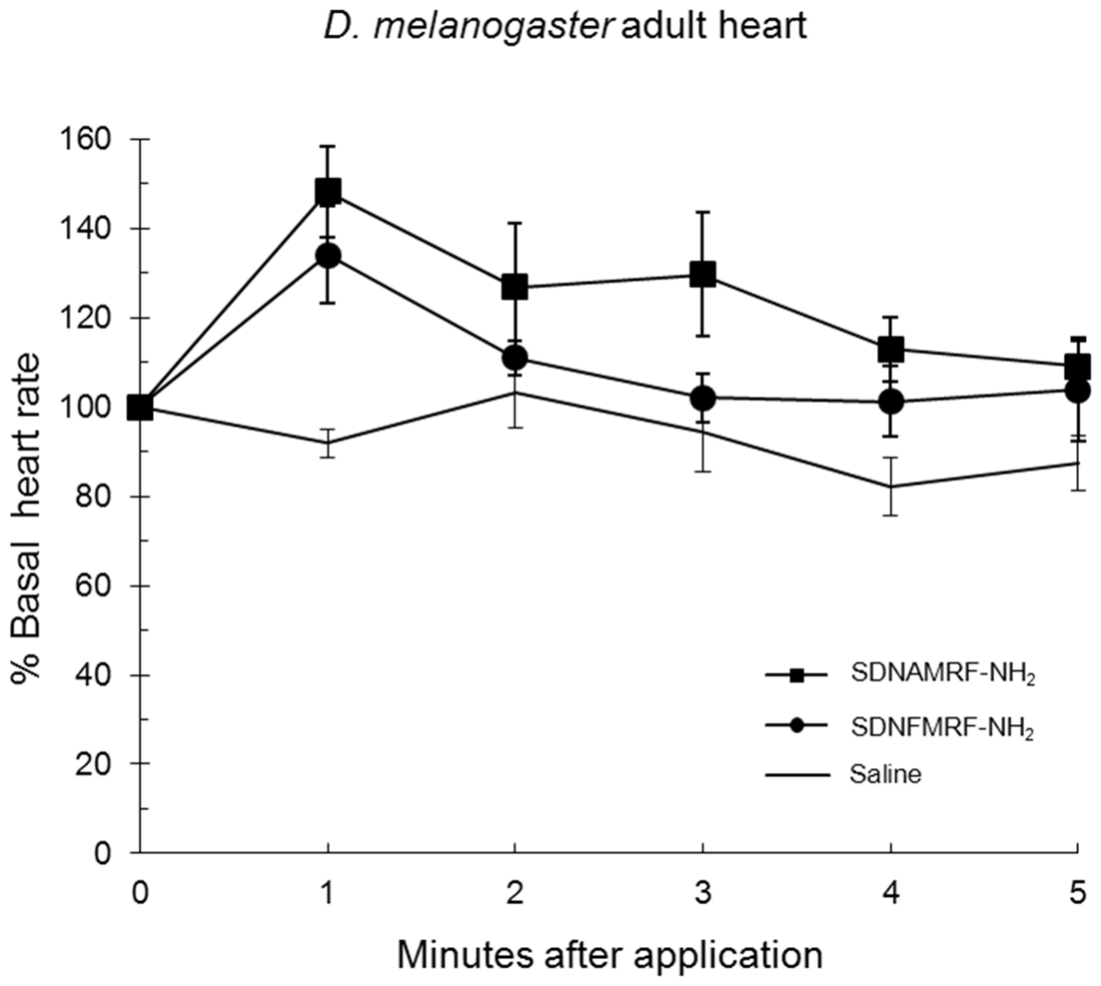
SDNAMRF-NH_2_ applied to heart. The effect of the analog SDNAMRF-NH_2_ on heart rate is reported as mean values ± S.E.M.

Next, the effect of the analog SDNAMRF-NH_2_ was measured on heart rate at 1 µM. SDNAMRF-NH_2_ increased adult heart rate by 48±10%, a significant effect similar to SDNFMRF-NH_2_ (Figure 12, Table 14). The effect of the peptide analog was analyzed in adult because it is a developmental stage in which a robust response occurs which thus allows for subtle changes to be observed. In addition to increasing heart rate, SDNAMRF-NH_2_ binds FMRFa-R less well than SDNFMRF-NH_2_ binds the expressed receptor protein; EC_50_  =  102 nM versus 1.9 nM, respectively [10].

**Table 14:** Cardioexcitatory effects of a *D. melanogaster* FMRF-NH_2_ analog a, b.

		% increase in heart rate	
		Adult	p value
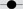	SDNFMRF-NH_2_	51±12	0.0006
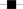	SDNAMRF-NH_2_	48±10	0.0003
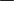	Saline	–13±2	

a The effects of SDNAMRF-NH_2_ on adult heart are reported as mean values ± S.E.M. calculated as percent increase in heart rate with p values relative to saline.

b Basal heart rate is equal to 100%.

### SDNAMRF-NH_2_ docked to FMRFa-R and applied to heart: Conclusions

Even though F→A substitution in SDNAMRF-NH_2_ caused it to lose important anchoring contacts made by the aromatic F1, the peptide had a docked pose very similar to SDNFMRF-NH_2_ (Figures 6B and 11, Tables 8 and 13) and was cardioexcitatory (Table 14). This is in contrast to AMRF-NH_2_ (Figure 5, Tables 5 and 6) in which F→A substitution caused major changes in contact sites and backbone shape, resulting in weaker ligand-receptor interactions. The ability of SDNAMRF-NH_2_ to maintain a docked pose like its parent peptide and interact with FMRFa-R to elicit a similar activity was consistent with the importance of the N terminus in docking and activity. In addition, SDNAMRF-NH_2_ maintained the contact sites predicted as critical for activity at FMRFa-R made by all five FMRF-NH_2_-containing peptides and FMRF-NH_2_, but not made by AMRF-NH_2_.

## Discussion

This study addressed the roles of an identical C-terminal tetrapeptide yet unique N-terminal extensions in ligand-receptor interactions and activity. Although the FaRPs, N-terminally extended FMRF-NH_2_ peptides, are structurally and functionally conserved throughout the animal kingdom and influence critical physiological processes including cardiac contractility, little is known about how these ligands interact with a receptor protein. Understanding ligand-receptor interaction is required in order to target the mechanisms involved in signaling and modulate function. Multiple FMRF-NH_2_-containing peptides, with an identical C-terminal FMRF-NH_2_ but unique N-terminal structure, are encoded in a single precursor. Although the precursor organization may contribute to the diversity of FaRP messengers available to regulate physiology, prior to this report, an investigation of what role the C-terminal tetrapeptide and N-terminal structure played in signaling had yet to be done.

The FMRF-NH_2_-containing peptides encoded in the *D. melanogaster* FMRFa gene, PDNFMRF-NH_2_, SDNFMRF-NH_2_, DPKQDFMRF-NH_2_, SPKQDFMRF-NH_2_, and TPAEDFMRF-NH_2_, are expressed and their structures confirmed. A receptor protein, FMRFa-R, representative of rhodopsin family A GPCRs was identified to which the peptides bind; EC_50_  =  2.0 nM (DPKQDFMRF-NH_2_), EC_50_  =  2.8 nM (TPAEDFMRF-NH_2_), EC_50_  =  1.9 nM (SDNFMRF-NH_2_), EC_50_  =  2.5 nM (SPKQDFMRF-NH_2_), and EC_50_  =  1.8 nM (PDNFMRF-NH_2_) [9], [10]. Past studies analyzed the effects of some or all of these peptides in a bioassay; yet no report to date investigated the SAR of the structurally-related ligands relative to their interactions with a single receptor protein [35]–[40]. In this study, the SAR of the five *D. melanogaster* FMRF-NH_2_-containing peptides, the C-terminal FMRF-NH_2_, and analogs were explored through docking, binding, and bioactivity using an assay reminiscent of the work done to characterize FMRF-NH_2_, the first FaRP identified [1], which allowed the effects of the peptides in multiple developmental stages to be analyzed.

The results of this study show F1 in FMRF-NH_2_ docked with identical, strong contact sites on TM3 in all five FMRF-NH_2_ peptides and FMRF-NH_2_, in agreement with its role as an anchor for ligand-receptor interactions. The contact sites, F197, S193, Q204, and N379, were made by all five FMRF-NH_2_ peptides and FMRF-NH_2_; this suggests the combination of these four contacts may play a role in binding to and/or activation of FMRFa-R. A notable difference in the interactions of the peptides versus the C-terminal structure alone was FMRF-NH_2_ made a few contacts with TM4, TM5, and TM6 to span the binding pocket; in contrast, the five naturally-occurring FMRF-NH_2_ peptides made extensive contacts with those TMs.

FMRF-NH_2_ made no predicted contacts with ECLs, yet, the five FaRPs would be expected to make numerous ECL contacts; all positioned a positively-charged residue near TM3 to cation pi-bond with aromatic residues on ECL1. These observations support the hypothesis; the C terminus makes extensive, strong ligand-receptor contacts, yet the N-terminal extensions influences docking. Furthermore, based on this study, the FMRF-NH_2_ peptides are anticipated to differ in ECL contacts; PDNFMRF-NH_2_ and SDNFMRF-NH_2_ positioned P or S and R for ECL contacts, DPKQDFMRF-NH_2_ and SPKQDFMRF-NH_2_ docked K, D, Q, and the C-terminal -NH_2_ in positions which would be accessible to the ECLs, and TPAEDFMRF-NH_2_ positioned T, P, R, and the C-terminal –NH_2_ for ECL contacts; these are distinctions between the peptides and how they interact individually with FMRFa-R.

These docking results were further supported by binding data and the bioassay which demonstrated the tetrapeptide and the five FMRF-NH_2_ peptides were cardioexcitatory. Docking demonstrated SDNAMRF-NH_2_ made contacts with FMRFa-R similar to how SDNFMRF-NH_2_ interacted with the receptor which was consistent with the bioassay in which the analog was cardioexcitatory. These results do not appear to agree with peptide-receptor binding; however, the disparity may lie in the ligand concentration used in the tissue-based bioassay versus EC_50_ values. In addition, these are two very distinct ways of assessing peptide-protein interactions involving assumptions not the least of which is the expressed receptor protein has the same structure as the native, naturally-occurring protein, which would require the correct and numerous processing events including post-translational modifications to occur. Also, the environment in which the receptor protein is expressed is not the same in heart tissue versus an expression system such as Chinese hamster ovary cells [10].

The effects of the naturally-occurring peptides and the analogs on heart rate were consistent with the overall docking results; the ligands made significant and strong, albeit unique contacts with the receptor. The relationship between the docking data and bioassay results must be further explored; there were similarities but uniqueness in the contacts between the peptides and the receptor, yet all five FaRPs elicited what appeared to be the same cardioexcitatory response. To more thoroughly consider the results the effects of the peptides on heart rate were based on the statistical analysis of the magnitude of the maximal response in amplitude at one concentration, 1 µM, which concluded the effects of all five FaRPs were the same. An additional output from this bioassay was the time course over which the peptides influenced heart rate after application (Figures 9, 10, and 12). Taking amplitude and time course into account, the data suggest testing over a range of ligand concentrations and/or additional experimental paradigm and recording devices may yield a more complex set of responses reflective of the differences in ligand docking. The measurement of the effects at one concentration in a single tissue-based bioactivity may not completely reflect ligand-receptor docking results. Nor does one bioassay represent the totality of physiological response(s) in a whole animal in its environment; rather it provides insight into the SAR required to elicit a response(s) due to a ligand acting through a peptidergic signaling pathway.

The fact that activity was observed in three different developmental stages is indicative of these structurally-related peptides, which are widely distributed in phylogeny, playing significant roles in physiology. The data collected provided insight into the design of an analog to test the hypothesis; the docking, receptor binding, and bioassay data for SDNAMRF-NH_2_ are in agreement with the influence of the N-terminal structure in ligand-receptor interactions and activity. The molecular structure and function approach in this study demonstrates the importance of the identical C-terminal tetrapeptide, but the influence of the unique N-terminal extensions in conferring docking and activity, which is critical insight needed in deciphering the mechanisms involved in FaRP signaling to target physiology.
